# Supplementary material for: EU protected area network did not prevent a country wide population decline in a threatened grassland bird
Source: PeerJ. 2018 Jan 23;6:e4284. doi: 10.7717/peerj.4284 (PMC5786059; doi:10.7717/peerj.4284)
Supplement: Supplemental Information 1 [file peerj-06-4284-s001.docx]

**S1. Additional description of the survey areas and population estimates**.

Little bustard breeding population estimates are aimed at adult males, because females are too inconspicuous to be detected in workable numbers (*e.g.* Silva *et al*. 2006; 2010).

The 2003-2006 national little bustard survey was aimed at three different types of areas:

- Special Protection Areas(SPAs) or Important Bird Areas (IBAs) for steppe birds, classified for their conservation importance to farmland birds.
- Outside SPA 5 x 5 km areas within 10 x 10 km UTM quadrats whose land surface was covered by more than 40% of open agricultural and pastoral land area (representing potential habitat for the species) based on information from Corine Land Cover 2000
- Prospection of previously unknown areas, not matching previously selected areas, dominated by open extensive agricultural landscapes. These areas when presenting low breeding densities were pooled to the non- SPA areas.

The breeding population was calculated for each SPA and for the area outside SPA (Table S1). We then extrapolated the mean density obtained from the respective survey points within and outside SPA to the total area of potential habitat in each class. The national male population estimate is the sum of population estimates within and outside SPA. Not all IBAs classified for farmland bird conservation were subsequently classified as SPA (two cases). Because these IBAs were not subjected to any sort of management, for the the purpose of this work, they were pooled to non-SPA areas.
