# Supplementary material for: EU protected area network did not prevent a country wide population decline in a threatened grassland bird
Source: PeerJ. 2018 Jan 23;6:e4284. doi: 10.7717/peerj.4284 (PMC5786059; doi:10.7717/peerj.4284)
Supplement: Table S1 — Results of the two national surveys, presenting the mean, minimum (min) and maximum (max) total male estimates for SPA and non-SPA, also indicating the proportion of variation and difference of number of males. [file peerj-06-4284-s002.docx]

**Table S1. Results of the two national surveys**, presenting the mean, minimum (min) and maximum (max) total male estimates for SPA and non-SPA, also indicating the proportion of variation and difference of number of males.
