# Supplementary material for: EU protected area network did not prevent a country wide population decline in a threatened grassland bird
Source: PeerJ. 2018 Jan 23;6:e4284. doi: 10.7717/peerj.4284 (PMC5786059; doi:10.7717/peerj.4284)
Supplement: Table S2 — Results from the 2003–2006 and 2016 SPA surveys, indicating the mean, minimum (min) and maximum (max) male density and male population estimates. The trend (considering stable populations with variations up to 10%), proportion of population variation and difference in number of males between surveys for each SPA are also presented. [file peerj-06-4284-s003.docx]

**Table S2. Results from the 2003-2006 and 2016 SPA surveys**, indicating the mean, minimum (min) and maximum (max) male density and male population estimates. The trend (considering stable populations with variations up to 10%), proportion of population variation and difference in number of males between surveys for each SPA are also presented.
